# Improving the power of drug toxicity measurements by quantitative nuclei imaging

**DOI:** 10.1038/s41420-024-01950-3

**Published:** 2024-04-18

**Authors:** Alesya M. Mikheeva, Mikhail A. Bogomolov, Valentina A. Gasca, Mikhail V. Sementsov, Pavel V. Spirin, Vladimir S. Prassolov, Timofey D. Lebedev

**Affiliations:** 1 Department of Cancer Cell Biology, Engelhardt Institute of Molecular Biology, Russian Academy of Sciences, 32 Vavilova str., Moscow, 119991 Russia; 2 Moscow Institute of Physics and Technology (National Research University), 9 Institutskiy per., Dolgoprudny, Moscow Region 141701 Russia; 3 Center for Precision Genome Editing and Genetic Technologies for Biomedicine, Engelhardt Institute of Molecular Biology, Russian Academy of Sciences, 32 Vavilova str., Moscow, 119991 Russia

**Keywords:** Cell death, Targeted therapies, Phenotypic screening

## Abstract

Imaging-based anticancer drug screens are becoming more prevalent due to development of automated fluorescent microscopes and imaging stations, as well as rapid advancements in image processing software. Automated cell imaging provides many benefits such as their ability to provide high-content data, modularity, dynamics recording and the fact that imaging is the most direct way to access cell viability and cell proliferation. However, currently most publicly available large-scale anticancer drugs screens, such as GDSC, CTRP and NCI-60, provide cell viability data measured by assays based on colorimetric or luminometric measurements of NADH or ATP levels. Although such datasets provide valuable data, it is unclear how well drug toxicity measurements can be integrated with imaging data. Here we explored the relations between drug toxicity data obtained by XTT assay, two quantitative nuclei imaging methods and trypan blue dye exclusion assay using a set of four cancer cell lines with different morphologies and 30 drugs with different mechanisms of action. We show that imaging-based approaches provide high accuracy and the differences between results obtained by different methods highly depend on drug mechanism of action. Selecting AUC metrics over IC50 or comparing data where significantly drugs reduced cell numbers noticeably improves consistency between methods. Using automated cell segmentation protocols we analyzed mitochondria activity in more than 11 thousand drug-treated cells and showed that XTT assay produces unreliable data for CDK4/6, Aurora A, VEGFR and PARP inhibitors due induced cell size growth and increase in individual mitochondria activity. We also explored several benefits of image-based analysis such as ability to monitor cell number dynamics, dissect changes in total and individual mitochondria activity from cell proliferation, and ability to identify chromatin remodeling drugs. Finally, we provide a web tool that allows comparing results obtained by different methods.

## Introduction

Large-scale drug screens provide valuable data for understanding drug mechanisms of action [1, 2], cancer cell vulnerabilities [3], development of novel drugs [4, 5] and drug repurposing [6]. The ability to kill specific cancer cells is a conventional indicator of anticancer drugs efficacy, however studies often rely on different methods to measure cell viability. Thus, several largest datasets provide drug toxicity data measured by methods, which rely on NADH activity, ATP or protein levels: Genomics of Drug Sensitivity in Cancer (GDSC) uses resazurin and CellTiter-Glo [3], Cancer Therapeutics Response Portal uses CellTiter-Glo [1], and NCI-60 uses sulforhodamine B assay [7]. These methods rely on indirect measurement of drug toxicity, and sometimes can be unreliable because drugs may influence cellular metabolic activity or protein levels without change in cells quantity [8–10]. Direct counting-based methods, such as trypan blue dye exclusion, are used to identify number of surviving cells. However, such methods constitute a laborious task for an operator, may give non-reproducible results and are not suitable for large-scale studies. Novel drug screens utilize more direct approaches such as measurement of DNA-barcoded cells in PRISM study [11] or real-time measurements of cell occupied area by IncuCyte or xCELLigence [12, 13]. Data obtained by different methods may have poor agreement [14, 15] due to changes in cell metabolism [8], adhesion, cell size and morphology [12], or drug influence on substrate used for measurement. Optimizing the consistency between NADH or ATP-based assays, measurements of cell area or LIVE/DEAD assays was addressed by numerous studies, providing either protocol optimizations, metric selection or method combination [16–19].

Advances in microscopy and image processing algorithms led to new high-content screening methods using fluorescence microscopy [20], that also allow viable cell counting [21–26]. Microscopy provides the most direct approach to measure cell proliferation and drug toxicity [21], as it does not require extensive processing of cells, such as trypsinization and cell lysis, and highly customizable by the use of fluorescent stains and proteins which allows measurement of cell cycle and cell death [27], protein activity [28–30], cell differentiation [31], metabolite levels and cellular morphology [32]. However, accurately segmenting single cells or nuclei is still a difficult task due to different growth patterns and drug-induced morphological changes.

Although relations between most common cell viability measurement methods were vastly explored before, there is still little data on how cell viability measurements using imaging-based methods correspond with other cell viability assays. Such data is essential for reliable integration of drug screen data from studies that used different readout methods and for selecting the appropriate study design. Thus, the main aim of this study was to compare cell viability measurements made by XTT colorimetric assay, which is similar to MTT and WST, but does not require a solubilization step, trypan blue dye exclusion and quantitative imaging to provide proper solutions for integrating data obtained by different methods. To determine number of cells by fluorescence microscopy we stained cell nuclei with Hoechst-33342 or utilized cells with continuous expression of fluorescent H2B-mRuby protein that allows automated nuclei counting. Since drug mechanisms of action and cell morphology can influence imaging results [33] we utilized different anticancer drugs and cancer cell lines. Here we explore how the mechanisms of action for different drugs affect differences in viability measurements performed by different methods and whether the results depend on a cell type used. All data is available through a web ShinyApp https://lebedevtdeimb.shinyapps.io/Mikheeva2023/.

## Results

### XTT measurements significantly differ from quantitative nuclei imaging

**Fig. 1:**
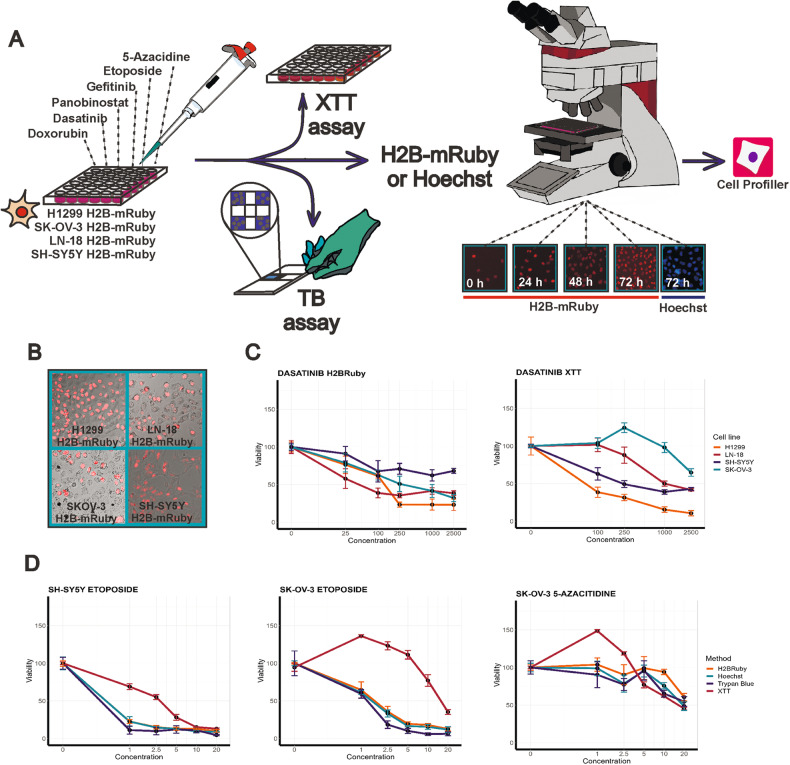
Cell viability measurement by different methods. **A**. Experiment design scheme. TB- trypan blue exclusion assay. **B** Representative images of cells, expressing H2B-mRuby protein (red). **C**, **D** Dose-dependent changes in cell viability measured at 72 h after drug treatment by XTT, trypan blue exclusion and nuclei counting using Hoechst staining or H2B-mRuby cells. Data presented as means and SD values for four repeats. Data was grouped by different cell lines for a single method (**C**) or by different methods for a single cell line (**D**). Concentrations are provided in logarithmic scale. Dasatinib was used in 25–5000 nM range, etoposide and 5-azacytidine in 0.25–20 µM range. All data were normalized on cell viability measurements for cells mock-treated with 0.1% DMSO.

To determine differences between some assays for measuring cell viability cancer cell lines of various origins: lung adenocarcinoma H1299, glioblastoma LN-18, ovarian adenocarcinoma SK-OV-3 and neuroblastoma SH-SY5Y were treated with different inhibitors and measured cell viability using four different methods (Fig. 1A). We used previously established H1299 and SH-SY5Y cells with H2B-mRuby expression [30] and introduced H2B-mRuby marker to LN-18 and SK-OV-3 cells by lentiviral transduction. Since not all cell population can be uniformly transduced (Figs. 1B and S1), which may affect assay results, we also used imaged-based counting using nuclei staining with Hoechst (Fig. 1A). Across different experiments the percentage of H2B-mRuby positive nuclei was stable and varied for H1299 cells between 93 and 97%, for SK-OV-3 between 60 and 76%, for SH-SY5Y between 55 and 68%, and for LN-18 between 26 and 34% (Fig. S1). Trypan blue dye exclusion assay (TB) allowed us to determine the exact quantity of viable cells and was later used as a benchmark. In order to account for different cell death mechanisms induced by anticancer drugs we selected six commonly used drugs with various mechanisms of action: doxorubicin, etoposide, dasatinib, gefitinib, panobinostat and azacitidine (5-Aza) (Table S1).

The difference between measurements obtained by different methods varied depending on a cell line and drug used. For example, the difference between SK-OV-3 and H1299 sensitivity to dasatinib was much higher when measured by XTT than using H2B-mRuby nuclei counting (Fig. 1C). The XTT assay produced exaggerated cell viability values in contrast to the results obtained by other methods, as observed for all cell types treated with etoposide or 5-Aza (Fig. 1D). However, in the case of 5-Aza, the difference in response was observed primary by non-toxic drug concentrations were XTT measurement provided higher readouts than in DMSO control (zero drug concentration). Data for all comparisons can be viewed using ShinyApp https://lebedevtdeimb.shinyapps.io/Mikheeva2023/.

### Variance in IC50 values is caused by combination of particular drug and method

**Fig. 2:**
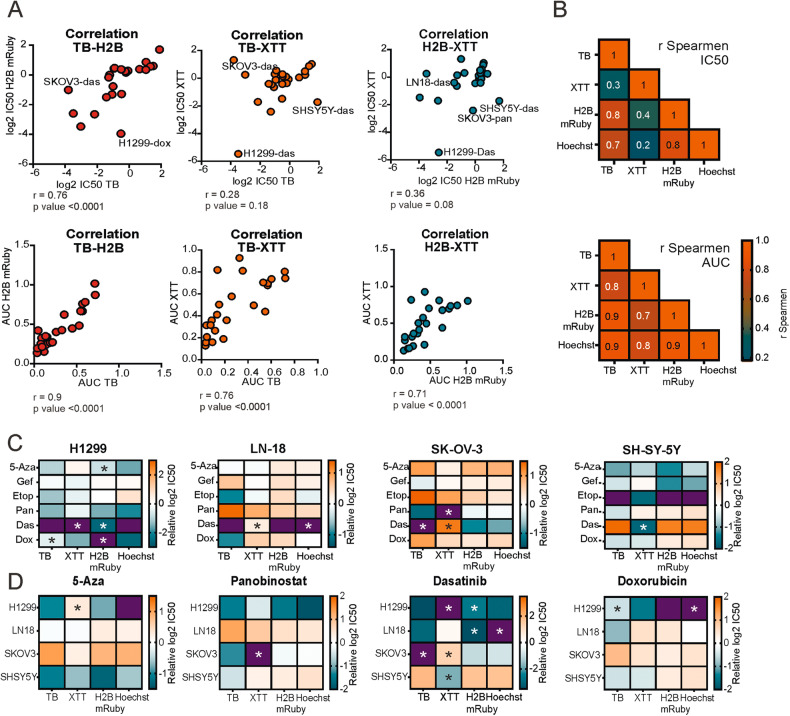
Correlation of IC50 and AUC between different assays. **A** Dot plots comparing IC50 and AUC values for different methods. Each point corresponds to cells treated with particular drug, most prominent outliners are annotated. Spearman’s correlation R and *p*-values are provided under each graph. H2B- values obtained by nuclei counting using H2B-mRuby, TB- trypan blue exclusion assay, das- dasatinib, dox- doxorubicin, pan- panobinostat. **B** Heatmap of pairwise Spearman’s correlations for IC50 and AUC values obtained by different methods. **C**, **D** Log2 IC50 differences heatmaps. For each cell lines and drug respective IC50 values were normalized on mean IC50 value for that drug across all cell lines. IC50 values were grouped by cell lines (**C**) or by drug (**D**). Color shows log2 difference between IC50 value and the mean IC50 for that drug and thus shows how sensitive particular cell line to a drug. Differences between IC50 value and the mean IC50 higher than fourfold are marked as purple. Stars highlight outliner cases when the differences between IC50 value and the mean IC50 of drug (**C**) or cell line (**D**) are more than twofold.

To quantitatively compare cell viability data we calculated IC50 (as concentration at which cell viability is reduced to 50%) and AUC values and then examined correlations between values obtained by different methods (Table S2). Correlation between IC50 values obtained from TB assay and H2B-mRuby or Hoechst nuclei counting were significant (*r* = 0.76 and 0.72; *p* values < 0.0001) (Figs. 2A, S2, and S3). Correlations between XTT and TB or H2B-mRuby assays were not significant (*r* = 0.28 and 0.36; *p* values > 0.08). However, when we compared the AUC values, correlations between each method pair were significant (Fig. 2B), even for TB and XTT assays (*r* = 0.76; *p* value < 0.0001), which had the weakest correlation for IC50 values (Fig. 2A). In general, comparing AUC values dramatically improved correspondence between data obtained by different methods (Fig. 2B). To investigate whether the cell origin or drug type drives the differences between IC50 values for selected methods, we compared differences in IC50 values for each treatment with mean IC50 values for a particular drug (Fig. 2C). As the result, we observed that XTT results had the highest number of identified outliers (at least twofold change in IC50) (Fig. 2C, D). Seven out of eleven outliners among all drugs and cell lines were for dasatinib, suggesting that particular method-drug combinations have higher influence on differences between measurements than the cells origin. We had not detected any outliners for AUC values, meaning that AUC values have less dependency on a method used to determine cell viability (Figs. S2 and S3).

**Fig. 3:**
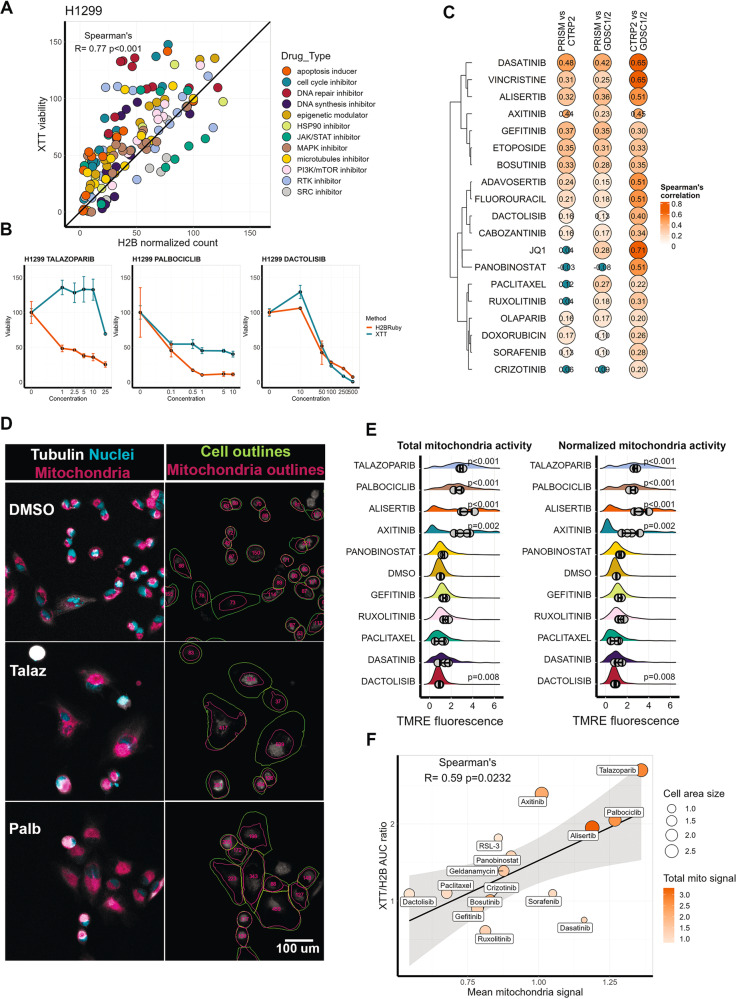
Drugs affect XTT measurements depending on their mechanism of action. **A** Correspondence between normalized cell viability of H1299 cells measured by XTT and H2B-mRuby imaging. Each dot represents the mean values between three repeats for each drug concentration. Cell viability values were normalized on values for cells mock-treated with DMSO. **B** Dose-dependent response of H1299 to talazoparib (1–25 µM), palbociclbib (0.1–10 µM) and dactolisib (10–500 nM). **C** Spearman’s correlations of AUC values between PRISM GDSC1/2 and CTRP2 datasets. Circle area is reverse proportional to correlation *p* value. **D** Staining and segmentation of H1299 cells treated with DMSO, palbociclib (Palb) or talazoparib (Talaz) for 72 h. In live cells tubulin was stained by Tubulin Tracker DeepRed (gray), nuclei with Hoechst-33342 (blue), and mitochondria with TMRE (magenta). Cell borders and areas occupied by mitochondria in each cell were determined by Cellpose and CellProfiler software. Total mitochondria signals (integrated TMRE fluorescence) for each cell are indicated by numbers. **E** Distribution of integrated TMRE signal per cell (total mitochondria activity) and TMRE signal per cell normalized to area occupied by mitochondria (normalized mitochondria activity) in H1299 cells treated with drugs or DMSO for 72 h. For each drug data for two toxic drug concentrations (indicated in Table S1) was combined. Dots show mean values for each biological repeat (*n* = 4) and SD based on repeats is provided. For each repeat four automatically selected imaging fields were analyzed. On average 650 cells were used to calculate each distribution. P-values were calculated using Mann–Whitney test by comparing mean values for each image with DMSO. **F** Correlation between the ratio of AUC obtained from XTT assay measurements to H2B-mRuby imaging and drug effect on mean mitochondria activity normalized to area occupied by mitochondria. Size of each dot is proportional to mean cell area as calculated by CellProfiler and color is proportional to total mitochondria activity induced by a drug.

Since drugs had significant effect on measurement variation between methods, we additionally compared measurements obtained by H2B-mRuby imaging and an XTT assay using H1299 cells and a panel of 30 drugs with different mechanisms of action. Overall, the Spearman’s correlation between methods was significant (*r* = 0.77; *p* value < 0.0001) (Fig. 3A). The highest differences between methods were caused by cell cycle inhibitors palbociclib, alisertib and adavosertib, DNA-damage repair inhibitor talazoparib and PKC inhibitor staurosporine (Table S2). We observed that the highest differences between measurements were caused by lower drug concentrations that reduced cell proliferation by less than 50%. For some drugs, for example palbociclib or talazoparib, unlike for drugs such as dactolisib, XTT assay failed to detect increasing drug toxicity (Fig. 3B). Data for all 30 drugs can be viewed using ShinyApp https://lebedevtdeimb.shinyapps.io/Mikheeva2023/.

To verify that similar relationship between methods is relevant for high-throughput drug screens and not unique to our case we compared drug sensitivity data for the drugs from three databases: GDSC1/2 [3], CTRP [1] and PRISM [6]. Cell viability in GDSC1/2 and CTRP were measured by colorimetric or luminometric assays: resazurin and Syto60 in GDSC1, and CellTiter-Glo in GDSC2 and CTRP. In PRISM dataset cell lines were labeled by DNA-barcodes and then pooled drug assays were performed and cell proliferation was measured by sequencing and enumerating the number of DNA-barcodes. As expected GDSC1/2 and CTRP datasets had high correlation scores, however PRISM had considerably weaker correlations with both GDCS1/2 and CTRP (Table S3). Only 7 drugs had *R* > 0.3 when PRISM data was compared with GDSC1/2 and 5 drugs did not have significant correlation for one of the comparisons (Fig. 3C). Notably the weakest correlations were for panobinostat and ruxolitinib, which also demonstrated high difference between measurement methods in our study (Table S3).

### Difference in cell viability measurements depends on drug-induced mitochondrial activity

As other studies suggested the elevated results of cell viability assays can be caused by increased mitochondria activity or by drug directly affecting substrate [10, 34–37]. Thus, we selected 15 drugs with highest and lowest differences between XTT and H2B-mRuby measurements and treated H1299 cells. Each drug was used in two concentrations which inhibited cell proliferation, and 72 h after drug treatment we stained cells with tubulin and nuclei stains to measure cell morphology, and with potential-dependent TMRE fluorescent stain to measure mitochondrial activity. Cell morphology changes and mitochondria activity for single cells were calculated using combination of Cellpose [38] and CellProfiler [39] pipelines. Several drugs, such as palbociclib, talazoparib, alisertib and axitinib significantly increased cell size, area occupied by mitochondria (Fig. S4) and overall TMRE staining intensity (Fig. 3D, E). Moreover, we detected an increase in TMRE signal when it was normalized by area occupied by mitochondria (Fig. 3E), meaning that these drugs not only increase overall cell size and thus integrated mitochondria activity per cell, but also individual mitochondrial activity. We did not detect such increase in mitochondrial activity for cell treated with other drug at toxic concentrations, except for dasatinib, meaning that this effect is specific to certain drugs. TMRE fluorescence normalized by area positively correlated (*r* = 0.59; *p* value = 0.02) with ratios between AUC values obtained by XTT assay and H2B-mRuby imaging (Fig. 3F). We also tested the effect of drugs added in growth medium without cells on absorption in XTT assay due to drug absorption properties or interaction with XTT, but we did not detect any significant changes in the readouts. These data provide systematic verification that selective increase in mitochondria activity caused by certain drugs affects readouts by metabolic-based assays.

### H2B-mRuby imaging reveals different cell death dynamics

**Fig. 4:**
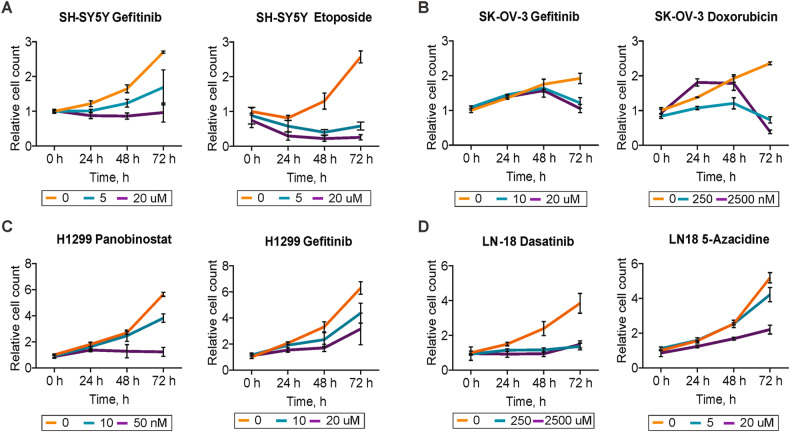
Measurement of cell proliferation dynamics using H2B-mRuby. Graphs show change in a number of identified nuclei for different drug concentration depending on time after start of the treatment of **A** SH-S5Y, **B** SK-OV-3, **C** H1299, and **D** LN-18 cells. Most representative drugs are shown for each cell line, data for other treatments can be viewed at https://lebedevtdeimb.shinyapps.io/Mikheeva2023/. Data presented as means and SD values based on four repeats. Nuclei counts were normalized to the nuclei counted before the start of the treatment.

The use of H2B-mRuby provides a non-invasive way to observe the dynamics of cell proliferation throughout the experiment. Although other methods like XTT or Hoechst staining also allow to measure dynamics they can significantly affect cellular processes and may cause additional toxicity. The dynamic analysis of cell viability revealed several types of responses based which couldn’t be predicted by endpoint analysis at 72 h. Our results highlight that interpretation of IC50 values at defined time point should take into account proliferation rate of particular cell line. For example, although gefitinib at 5 µM reduced SH-SY5Y cell numbers by two times, it failed to prevent cell proliferation (Fig. 4A) and actual proliferation inhibition occurred only at 20 µM. On the other hand, for SK-OV-3, which have slower proliferation rate, the two-fold drop in cell number at 72 h indicates full proliferation inhibition (Fig. 4B). In some cases, measuring cell number dynamics can help to distinguish drugs that actively kill cells and not just slow down proliferation. For example, we detected reduction in number of nuclei for SH-SY5Y treated with etoposide after 24 h and for SK-OV-3 treated with gefitinib or doxorubicin after 48 h (Fig. 4A, B). Similar relations were observed using other cell lines, irrespective of their proliferation rates. For example, even though gefitinib reduces overall number of H1299 cells, cell continue to proliferate in the presence of the drug (Fig. 4C), similar to SH-SY5Y (Fig. 4A). Dasatinib on the other hand fully inhibits proliferation of LN-18 cells (Fig. 4D) similar to effects of etoposide on SH-SY5Y (Fig. 4A). However, the use of fluorescent protein may be restricted by drug fluorescence, such as in case of 2500 nM of doxorubicin, which caused a false increase in cell numbers at 24 and 48 h due to high accumulation of fluorescent drug in cells (Fig. 4B). Cell proliferation dynamics for each treatment can be view using ShinyApp https://lebedevtdeimb.shinyapps.io/Mikheeva2023/.

### H2B-mRuby fluorescence can be used to identify chromatin remodeling drugs

**Fig. 5:**
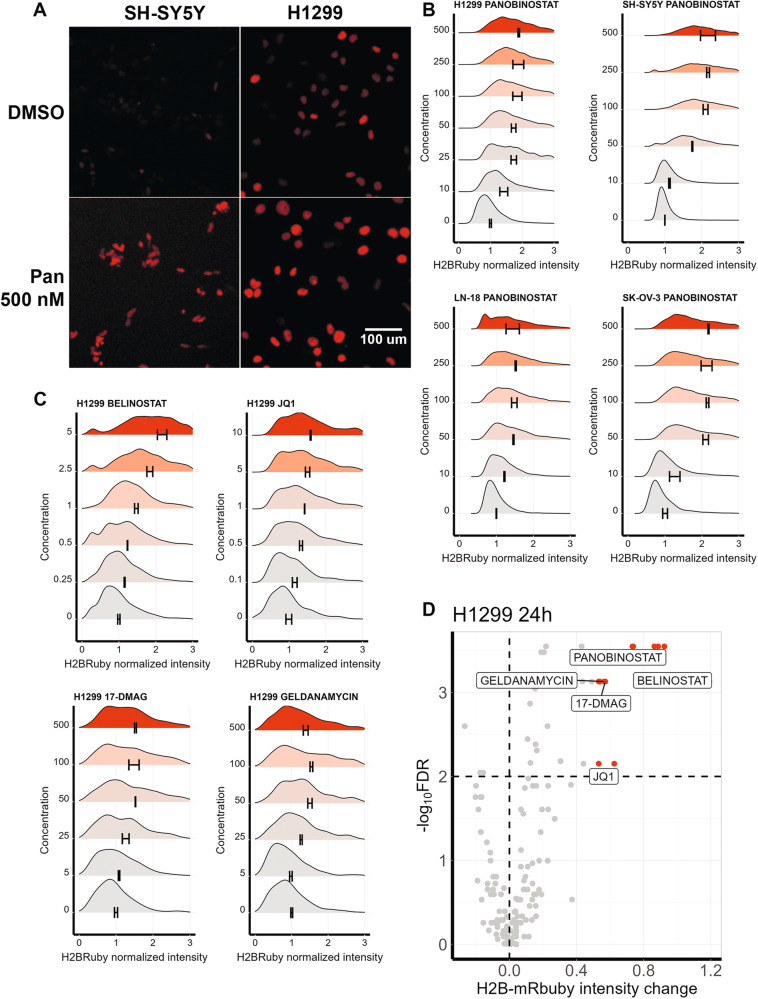
Chromatin remodulating drugs induce H2B-mRuby fluorescence. **A** Representative images of nuclei H2B-mRuby fluorescence in H1299 and SH-SY5Y cells treated with DMSO or 500 nM panobinostat (Pan) for 24 h. **B** Distribution of median H2B-mRuby fluorescence per nucleus for H1299, SH-SY5Y, LN-18 and SK-OV-3 cells treated with panobinostat for 24 h. H2B-mRuby fluorescence was normalized to cells treated with DMSO (zero concentration) and distribution was calculated based on average on 550 nuclei. On average three repeats were performed for each condition and 4 automatically selected fields were imaged. SD is indicated as +- range from mean based on mean fluorescence values for each repeat. **C** Distribution of median H2B-mRuby fluorescence per nucleus for H1299 cells treated with belinostat, JQ-1, 17-DMAG and geldanamycin for 24 h. **D** Volcano plot showing differential increase in H2B-mRuby fluorescence for H1299 cells treated with 30 different drugs for 24 h compared to DMSO-treated cells. Each dot represents mean fluorescence for a drug used in particular concentration (Table S4), maximum effects for statistically significant drugs are labeled. *P* values were calculated using Mann–Whitney test based on mean values for each image and then Benjamini-Hochberg correction for multiple testing was applied (FDR).

When we processed H2B-mRuby images, we also noted that several drugs caused an increase in H2B-mRuby fluorescence intensity. Across all cell lines, this increase was prominent when cells were treated with panobinostat and this effect was concentration-dependent (Fig. 5A, B). We hypothesized that increase in H2B-mRuby fluorescence may be due to increase in H2B-mRuby expression, which is controlled by human PGK promoter [40], after HDAC inhibition by panobinostat. Viral promoters often get silenced in cells and HDAC inhibitors are known to be able to reactivate HIV-1 gene expression during latent infection stage [41]. To verify, that this effect was caused by HDAC inhibition we additionally tested another HDAC inhibitor belinostat. It increased H2B-mRuby signal intensity in a similar manner as panobinostat (Fig. 5C). To check if this effect is specific to HDAC inhibitors we measured H2B-mRuby intensity under all 30 tested drugs for H1299 cells (Table S4). We found that well described chromatin remodeling drug JQ-1 that inhibits BET also significantly increased H2B-mRuby fluorescence (Fig. 5C, D). All other drugs, except HSP90 inhibitors geldanamycin and 17-DMAG, did not have significant effect on H2B-mRuby signal intensity (Fig. 5C, D). None of the drugs except doxorubicin were fluorescent by themselves in used concentrations as was tested on H1299 cells without H2B-mRuby (Fig. S5). For selected drugs that increased H2B-mRuby fluorescence we additionally measured their effect on expression of H2B-mRuby, hygromycin resistance gene and WPRE signal encoded by lentiviral vector using real-time PCR. Panobinostat, belinostat and JQ-1 increased expression of all measured genes at least two-fold (Fig. S6). HSP90 inhibitors 17-DMAG and geldanamycin had no effect on gene expression, meaning increase in H2B-mRuby fluorescence probably occurs due to increased protein stability or translation efficacy, but not because of epigenetic regulation. These findings suggest that a fluorescent protein expressed as a transgene can be additionally used to find drugs with chromatin remodeling properties. H2B-mRuby intensity distribution for each treatment can be viewed using ShinyApp https://lebedevtdeimb.shinyapps.io/Mikheeva2023/.

## Discussion

Our results show, that although XTT assay and imaging methods often produce different results, the use of AUC metric overall provides consistent comparisons. Although IC50 is a convenient metric which offers an easily interpretable value it should be used with caution when comparing values from different datasets, especially obtained by different methods. We propose that it is more reliable to compare either concentrations which reduce proliferation by more than 20% or use AUC metric. The limitations of IC50 calculation can be somewhat bypassed by considerable increase of the number of drug concentrations used in a test, however this can significantly increase the cost and time for large-scale tests. Our findings are consistent with the results of other studies, which show that AUC or other area-based metrics, like DSS, produce more reliable results, especially for prediction of drug sensitivity [42, 43].

The variance between measurements performed by different methods also depended on a treatment selection, especially for cytostatic drugs [19, 44], knockdown of some genes [45] or radiation exposure [10] as has been shown before. For example, palbociclib is known to induce cell size growth [46] and accumulation of mitochondria, thus resulting in false results obtained by methods relying on mitochondrial activity [35]. We showed that palbociclib as well as other cell cycle inhibitors not only induced mitochondria accumulation due to increased cell size, but also increased mitochondrial activity itself. This effect we observed not only for well-established cell cycle inhibitors such as palbociclib and alisertib, but also for PARP inhibitor talazoparib and VEGFR inhibitor axitinib. Since these inhibitors can induce DNA-damage response and lead to G2/M arrest [47, 48], increased cell size (Fig. S4) and senescence [49, 50] in some cell types, we assumed that senescent phenotype could be responsible for increased mitochondrial activity. However, we detected senescent cells only for cells treated with axitinib, and other drug which increased mitochondria activity failed to induce senescence (Fig. S7). There are several studies suggesting that increased cell size leads to higher mitochondria mass [51, 52]. We see that area occupied by mitochondria is larger in bigger cells (Figure S4), which can mean that mitochondria mass remains the same but they are more spread out in cytoplasm. In our experiments we also detected an increase of total mitochondria activity per cell (Fig. 3E), meaning either increase in mitochondria mass or activity of individual mitochondria. Although we cannot strictly distinguish between changes in mitochondria mass and activity of individual mitochondria, given previously published studies [51, 52], we think it is more likely that several processes happen as cells grow in size. Thus, as a cell grows mitochondria occupy larger area within the cytoplasm, increase their mass and activity of individual mitochondria. High-content imaging allows to accurately distinguish effects on cell viability as number of nuclei, area occupied by mitochondria, total mitochondria activity as integrated intensity per cell, and changes in individual mitochondria activity as signal normalized by area occupied by mitochondria in each cell. For example, dasatinib reduces cell size, which results in slightly decreased overall mitochondria signal, however dasatinib-treated cells had higher mitochondrial activity per occupied area, suggesting potentially different mechanisms of dasatinib action compared to similar inhibitors such as bosutinib [53]. The higher variance between methods readouts observed for dasatinib may connected to both decrease in mitochondria activity and cell size, but also due to cells forming tight clumps, which negatively affects nuclei segmentation accuracy.

One of the concerns with the use of H2B-mRuby for cell counting is that cells have heterogeneous levels of transgene expression, and since not all cells have detectable transgene expression the changes in the number of H2B-mRuby positive cells might not represent the changes in numbers of all cells. Also, the introduction of transgene might make transduced cell subpopulation more or less sensitive to a specific drug. However, even though our cell lines had varied percentage of H2B-mRuby positive cells (from 25 to 90%) and H2B-mRuby intensity, the results between nuclei counting with H2B-mRuby were highly consistent with nuclei counting using Hoechst staining or with results of trypan blue exclusion assay. The main drawback of using H2B-mRuby is the necessity of creating transgene cells, which might not be possible in case of ex vivo drug screens using patient-derived cells.

We describe several advantages of using H2B-mRuby: the ability to non-invasively record cell proliferation dynamics and find potential chromatin modulators. The proliferation dynamics can improve drug classification based on whether they prevent cell proliferation completely, reduce the initial cell numbers or allow cells to proliferate even at slower rates. We showed that H2B-mRuby intensity changes in response to chromatin remodeling drugs, such as HDAC and BET inhibitors. Several studies also suggested the possibility to use transgene expression to detect drugs that affect cell epigenetics. These approaches used cells with silenced GFP transgene and then drugs which affected epigenetic factors reactivated GFP expression, increasing the number of GFP positive cells [54–56]. We show that similar approach works even if introduced transgene was not completely repressed in the majority of cells, and expression of H2B-mRuby under PGK promoter can be used as a potential reporter for chromatin remodeling drugs. H2B-mRuby imaging or other genetically encoded fluorescent protein can be used as a high-through approach to identify drugs with chromatin remodeling capacity, however it should be verified by gene expression analysis, since some drugs such as HSP90 inhibitors can increase protein fluorescence without affecting gene expression.

In conclusion, modern imaging-based approaches provide several benefits to large-scale drug screens, such as higher cell counting accuracy, ability to measure cell proliferation dynamics, and perform additional measurement such as the use of H2B-mRuby fluorescence intensity as reporter for chromatin remodulation. We show that AUC metric provides more consistency when comparing cell viability results obtained by imaging methods with results of conventional assays. We also identify main reasons for measurement differences, such as increased cell size, induction of senescent phenotype or altered mitochondrial activity- factors, which should be considered for consistent integration of imaging data with existing large-scale drug screens.

## Methods

### Cell culture, lentiviral transduction and materials

All cell lines are not in the list of commonly misidentified cell lines that are controlled by the International Cell Line Authentication Committee. Table S1 contains list of reagents used for cell cultivation, cell densities used in experiments and growth conditions for each cell line. Cells were routinely checked for mycoplasma with Hoechst-33342 and DAPI staining, and to prevent mycoplasma contamination cells were treated with EZkillTM Mycoplasma Elimination Kit (HiMediaLabs) after defrosting. Lentiviral preparation was performed as described previously [30] using pLentiPGK Hygro DEST H2B-mRuby2 (Addgene #90236) [40] and then cells were transduced to achieve at least 25% transduction rate. To ensure that we generated stably transduced H2B-mRuby cells, cultured for two-three weeks to ensure stable levels of H2B-mRuby expression and then created a cryogenic stock of the cells. We used aliquots of cells from the same cryogenic stock and cultivated them no longer than 4–5 weeks after thawing. Cells expressing H2B-mRuby were then enriched using selection with 0.5 mg/ml hygromycin b (Sigma) and verified using fluorescence microscopy and flow cytometry (LSRFortessa flow cytometer, BD Biosciences). All materials used and their manufacturers are listed in Table S1.

### Drug treatment, AUC and IC50 calculation

Cells were seeded in indicated densities to 96-well plates for XTT and imaging, and in 48-well plates for trypan blue exclusion assay 24 h before drug treatment. All drug’s working solutions in DMSO were stored at −20 °C as aliquots in concentrations at least 1000× to a maximum concentration applied to the cells, all concentrations are listed in Table S1. On the same day before cell treatment these solutions were unfrozen, thoroughly mixed and checked for precipitates, each stock aliquot was unfrozen no more than 5 times. Then drugs dissolved in the growth medium as 10× stocks, and appropriate amount of DMSO was added to equalize DMSO concentrations for all treatments, and then drugs were added to the cells. DMSO concentration did not exceed 0.1% for all treatments. After 72 h incubation cell viability was analyzed using XTT, imaging or trypan blue exclusion assay. Trypan blue exclusion assay was performed manually by two independent researchers using Neubauer chamber. Prior to cell counting in Neubauer chamber cells were washed with PBS, trypsinized at 37 °C and 5% CO_2_ for 5 min and resuspended in complete medium. XTT assay was measured by 450 nm absorbance and 650 nm reference using Multiskan FC (ThermoScientific, USA) after 4 h incubation at 37 °C and 5% CO_2_, reference signal for each well and mean signal for wells containing only growth medium and XTT were subtracted before normalization. For XTT and trypan blue dye exclusion experiments were repeated three times. For nuclei staining 1 µg/ml Hoechst-33342 was added and then cells were incubated at 37 °C and 5% CO_2_ for 30 min before imaging. For H2B-mRuby imaging cells were imaged without any additional staining. Wells were treated independently two wells in the same experiment, which were repeated three times. For each well six fields were automatically selected with the same pattern for all wells. Number of nuclei for each image was calculated using CellProfiler pipeline and data was extracted using custom Python script. Then nuclei counts were averaged for each repeat between six imaging fields. All results were normalized to mean values of control treatment (no drug), control treatment was considered as 100% and complete absence of cells as 0%. IC50 values were calculated using four-variable non-linear regression in GraphPad Prism 9 software with top and bottom values set at 100 and 0. AUC values were calculated using trapezoidal rule in Python. For cell dynamics number of nuclei were also normalized to the number of nuclei at the start of experiment for each repeat. All drug treatment data is available for viewing via ShinyApp https://lebedevtdeimb.shinyapps.io/Mikheeva2023/.

### Cell staining and fluorescence microscopy

Cells were imaged on 96-well plates using motorized Leica DMI8 fluorescence microscope (Leica, Germany). For nuclei staining 1 µg/ml Hoechst-33342 was added, for tubulin and mitochondria imaging 1 µl of Tubulin Tracker™ Deep Red (Invitrogen, USA) and 100 ng/ml TMRE (Lumiprobe, Russia) were added, and then cells were incubated at 37 °C and 5% CO_2_ for 30 min before imaging. Hoechst-33342 was imaged with excitation 325–375 nm and emission in 435–485 nm, TMRE and H2B-mRuby were imaged with excitation 541–551 nm and emission in 565–605 nm, Tubulin Tracker™ Deep Red was imaged with excitation 590–650 nm and emission in 662–738 nm. For nuclei staining autofocus was performed using Hoechst-33342 images, for H2B-mRuby protein using bright field images, and for TMRE, tubulin, and nuclei staining. Plate layout and autofocus were done using LAS X software. For each well (biological repeat of drug treatment) four to six fields were automatically selected with the same pattern for all wells. Image quality control was performed manually by two researches and using custom Python scripts. Images with low quality or presence of optical obstacles (areas with high background signal and serum debris) were excluded from analysis. Senescence staining was performed 144 h after drug treatment using β-Galactosidase Staining Kit (Cell Signaling Technology, USA). Treatment with SCH772984 was used as a positive control to induce senescence in cancer cells [57].

### Nuclei and cell segmentation

Nuclei segmentation was performed in CellProfiler v4.2 [39]. First, we performed manual control of image quality to remove unfocused images or images with artifacts. Then images of Hoechst stained or H2B-mRuby2 labeled nuclei were corrected using *CorrectIllumination* module in CellProfiler. Correction function was calculated and applied for each image using *background method* with *fit polynomial smoothing*. To segment nuclei, we used *adaptive threshold strategy* with *Sauvola thresholding method*. Thresholding parameters for Hoechst-stained nuclei were selected the same for each cell line, as staining intensity was the same. For H2B-mRuby labeled nuclei parameters for *object diameter* and *lower threshold* varied depending on fluorescence intensity for each cell line.

For mitochondria activity measurements we segmented cells using combination of Cellpose v2 and CellProfiler v4 pipelines. First all images of Hoechst, TMRE and Tubulin Tracker were corrected using *CorrectIllumination* module in CellProfiler. Correction function was calculated and applied for each image using *background method* with *fit polynomial smoothing*. Then for better cell segmentation, we merged grayscale TMRE and tubulin images. These merged images were used for cell segmentation in Cellpose with cyto2 pre-trained model. To account for cells of different sizes we used two different object diameters for each image and for each image two masks were generated: for smaller cells using diameter 35 pixels and for big cells using diameter 150 pixels. Cellpose masks and other images were then loaded into CellProfiler, which was used for initial nuclei segmentation. Nuclei segmentation was also performed using two different expected object diameters: for small and big nuclei. Then four object sets (small cells, big cells, small nuclei and big nuclei) were combined using CellProfiler pipeline. We selected cells that have identified nuclei of respected size inside them, and then for each big cell we compared areas occupied by objects belonging to those cells. Based on that comparison we decided whether these are correctly identified big cells, or a misidentified group of smaller cells. To identify areas occupied by mitochondria we use images of TMRE staining and performed segmentation in Cellpose using cyto2 pre-trained model. Then we used CellProfiler to assign segmented mitochondria areas to each cell based on overlap of these objects, and measured TMRE intensities for each mitochondria area. Image analysis data was processed using custom Python scripts and visualized using ggplot2 in R and GraphPad Prism v9. Python code for Cellpose and CellProfiler pipelines are available on GitHub https://github.com/CancerCellBiology/Cell_count_methods.

### Drug screen database analysis

Drug response data for GDSC1/2 [3], CTRP [1] and PRISM [6] drug screens was downloaded from DepMap [58, 59] portal (https://depmap.org/portal/). Drug response was downloaded as drug AUC values for each cell line. First, we selected drugs present in GDSC2 dataset and then added data from GDSC1 for the drugs which were not present in GDSC1, then we selected drugs present in all GDSC1/2, CTRP2 and PRISM datasets. Next, we calculated Spearman’s correlation for each drug between each pair of datasets. For each pair we selected cell lines for which drug response data were present in both datasets. The average number of cell lines used to calculate correlation between datasets ranged from 267 to 464 cell lines. Drugs were clustered using Euclidean metrics and Ward2 hierarchical clustering, heatmap was generated using ComplexHeatmap R package [60]. Code used for data analysis and data visualization is provided on GitHub https://github.com/CancerCellBiology/Cell_count_methods.

### Real-time PCR

Primers were chosen based on the viral insertion sequence, ensuring a length of approximately 20 base pairs, absence of hairpins, and an annealing temperature of 57 °C. Primers for measuring H2B-mRuby expression were designed to amplify the part of the sequence were H2B and mRuby are fused to avoid amplifying endogenous H2B mRNA. The primer sequences are presented in Table S1. H1299 cells were cultivated at a density of 60,000 cells per well in a 6-well plate and treated with the IC50 concentrations of drugs, which lead to enhance nuclear fluorescence. After 24 h cells were lysed by TRIzol Reagent (Ambion by Life Technologies) for RNA extraction following the manufacturer’s guidelines. Subsequently, 5 μg of mRNA were used for the synthesis of cDNA. Real-time PCR was performed in triplicate using Maxima SYBR Green Supermix (Thermo Scientific, Waltham, MA, USA) and the CFX96 Real-Time System (Bio-Rad, Hercules, CA, USA). Expression data was normalized to the expression levels of human GAPDH.

### Statistical analysis

Mann–Whitney test and *t*-tests were performed using SciPy Python package and Benjamini-Hochberg correction for multiple testing was performed using statmodels Python package. Spearman’s correlation was calculated using GraphPad Prism 9 and SciPy Python package. Mean, SEM and SD values for cell viabilities were calculated in R and GraphPad Prism 9.

### Supplementary information

Supplemental Material

Table S1

Table S2

Table S3

Table S4

## Data Availability

All data are available for viewing via ShinyApp https://lebedevtdeimb.shinyapps.io/Mikheeva2023, and processed data are provided in supplementary tables, Python and R codes, and CellProfiler pipelines are available on GitHub https://github.com/CancerCellBiology/Cell_count_methods.
