# Supplementary material for: Improving the power of drug toxicity measurements by quantitative nuclei imaging
Source: Cell Death Discov. 2024 Apr 18;10:181. doi: 10.1038/s41420-024-01950-3 (PMC11026393; doi:10.1038/s41420-024-01950-3)
Supplement: Supplementary file 1 — Supplemental Material [file 41420_2024_1950_MOESM1_ESM.pdf]

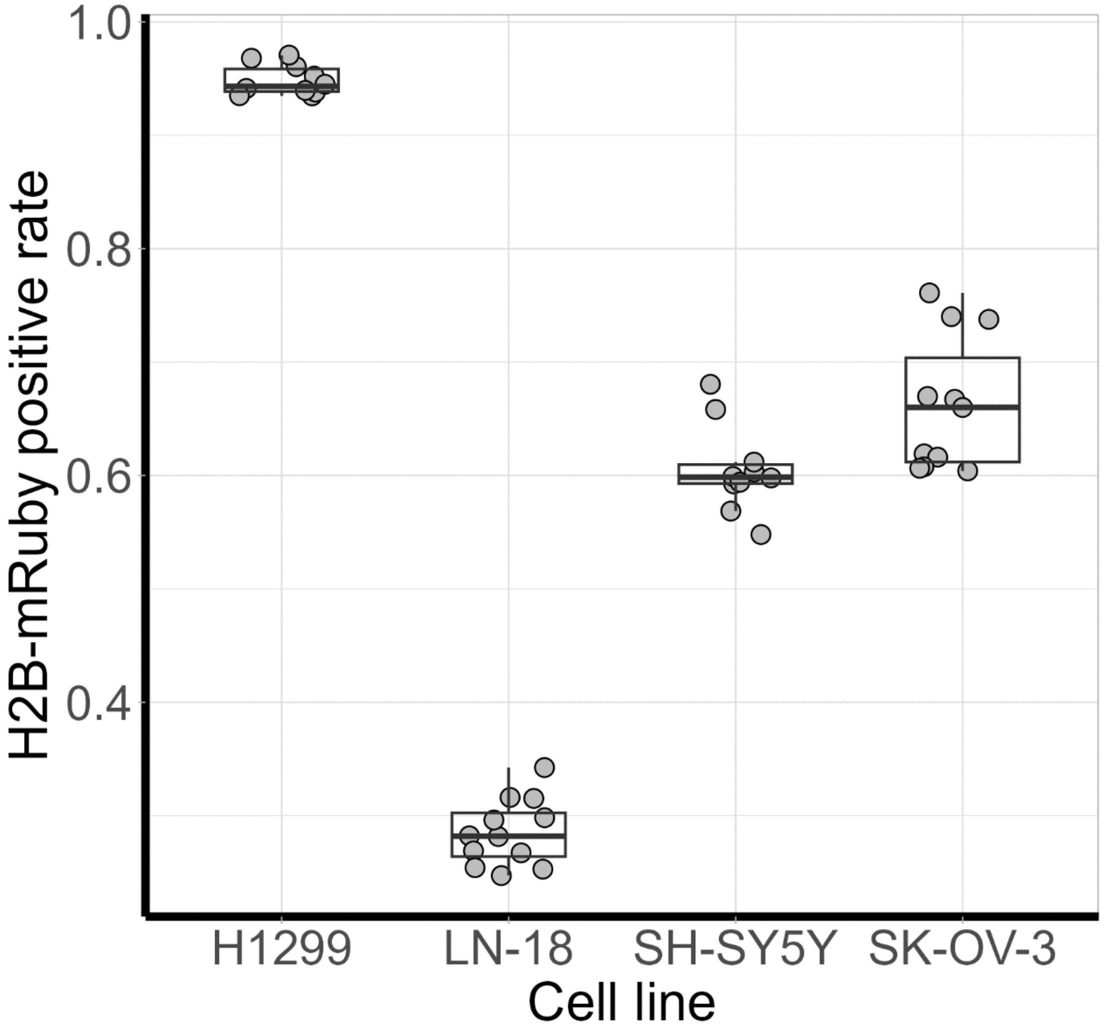

Figure S1. Rate of H2B-mRuby positive cells in each cell line. Each dot shows the rate of H2B-mRuby positive nuclei among nuclei identified by Hoechst staining in each separate experiment (different drug treatments and biological repeats). Box plots show mean values and 25, and 75 percentiles.

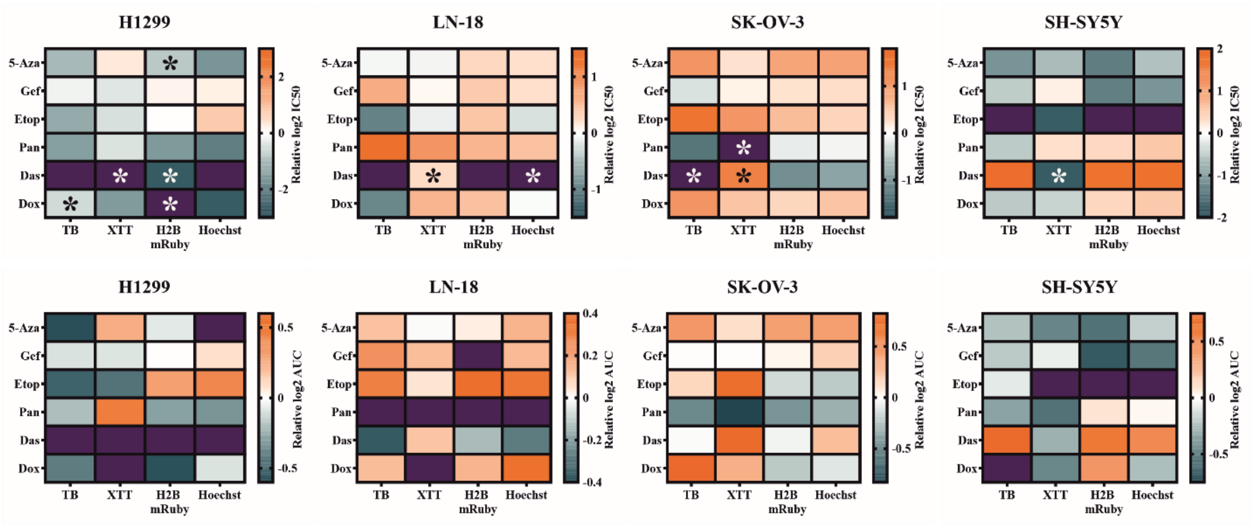

Figure S2. Drug IC50 and AUC differences heatmaps for cell lines. For each cell lines and drug respective log2 IC50 (top) and AUC (bottom) values were normalized on mean value for that drug across all cell lines. Values were grouped by cell lines**.** Color shows difference between value measured by particular method and the mean for that drug and thus shows how sensitive particular cell line is to a drug. Differences between IC50 value and the mean IC50 higher than 4-fold are marked as purple. Stars highlight outliner cases when the differences between IC50 value and the mean IC50 of drug are more than two-fold.

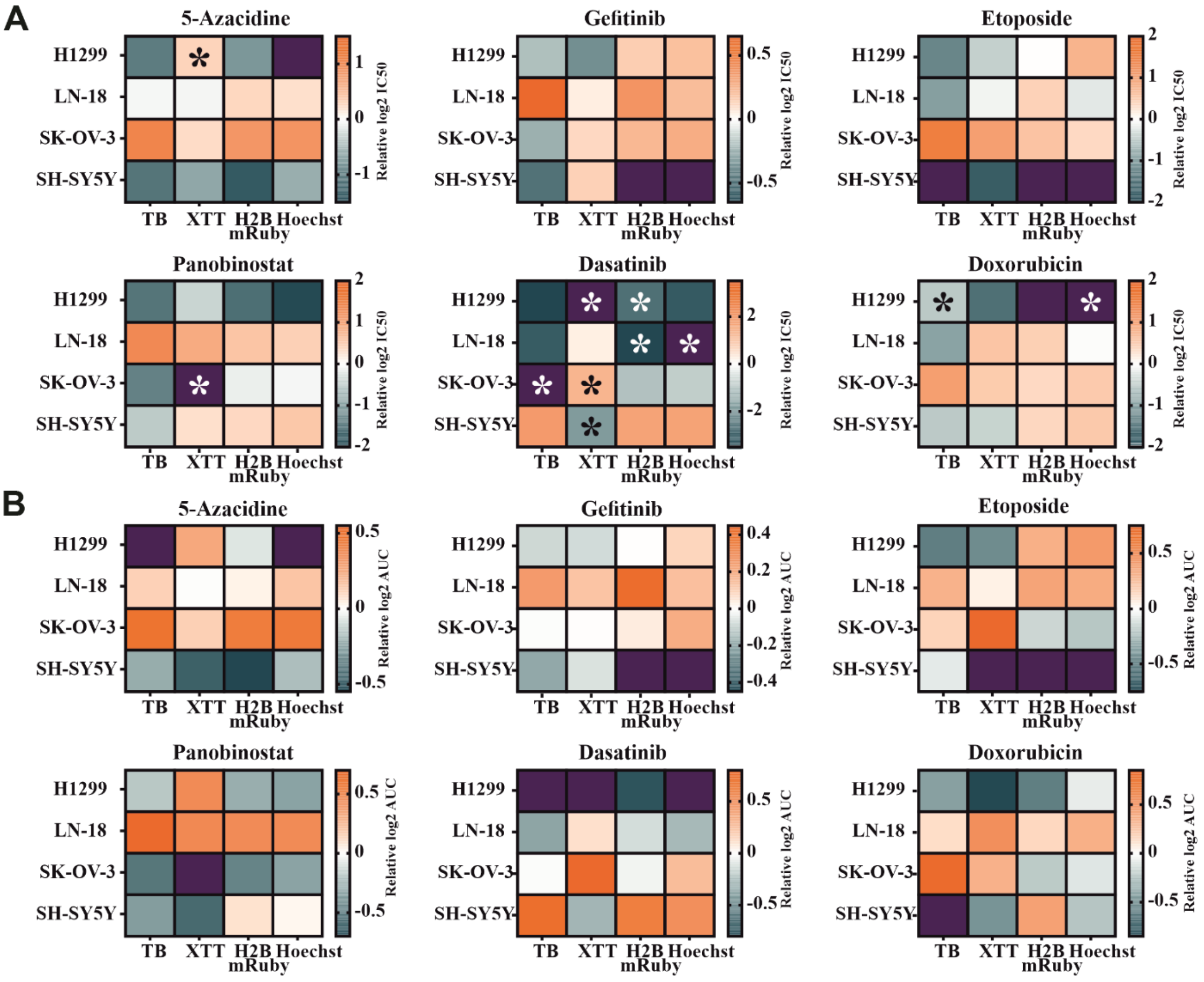

Figure S3. Drug IC50 and AUC differences heatmaps for drugs. For each cell line and drug respective log2 IC50 **(A)** and AUC **(B)** values were normalized on mean value for that drug across all cell lines. Values were grouped by drugs**.** Color shows difference between value measured by particular method and the mean for that drug and thus shows how sensitive particular cell line is to a drug. Differences higher than 4-fold are marked as purple. Stars highlight cases when the differences are more than two-fold.

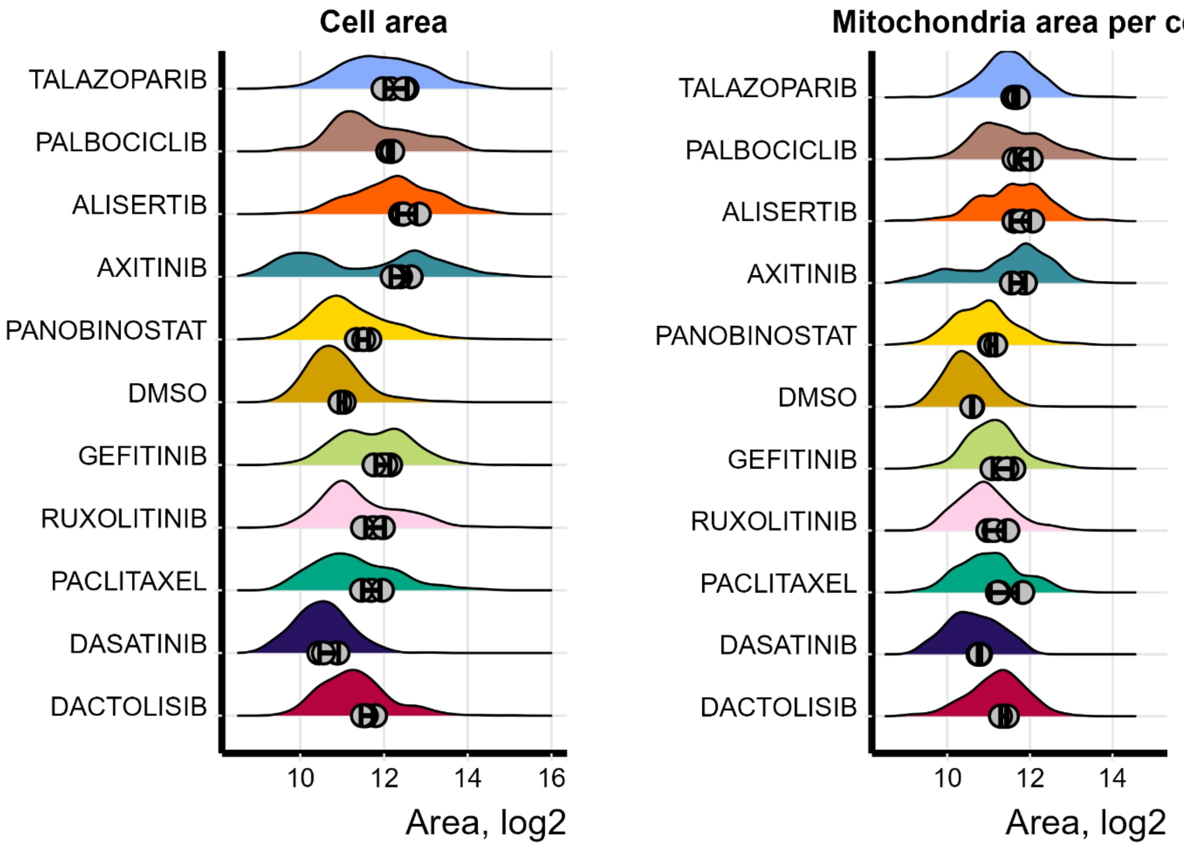

Figure S4. Distribution of cell area and mitochondria area per cell in H1299 cells treated with drugs or DMSO for 72h. For each drug data for two toxic drug concentrations (indicated in Table S1) was combined. Dots show mean values for each biological repeat (n=4) and SD based on repeats is provided. For each repeat four automatically selected imaging fields were analyzed. On average 650 cells were used to calculate each distribution. P-values were calculated using Mann-Whitney test by comparing mean values for each image with DMSO.

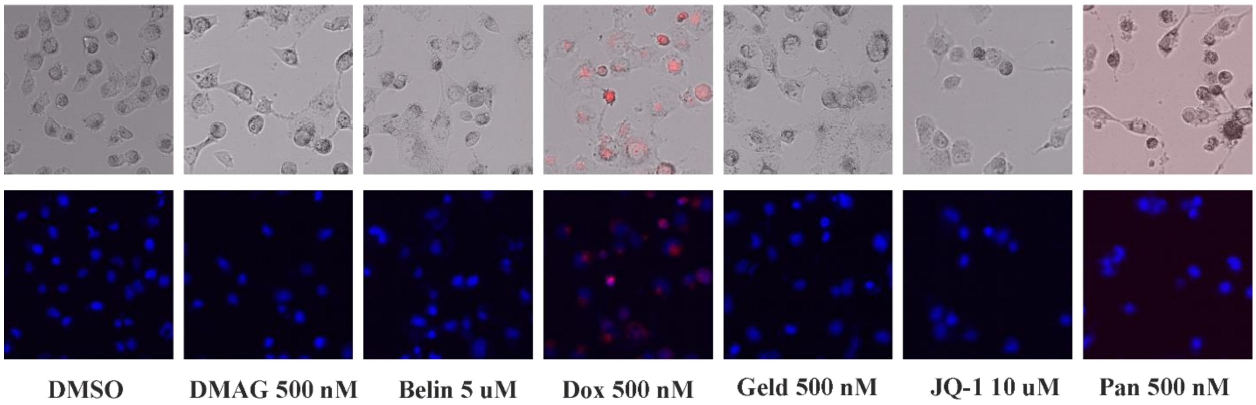

Figure S5. Images of drug fluorescence in H1299 cells. Representative images of drug fluorescence with 541-551 nm excitation and 565-605 nm emission (red) are shown. Upper row shows drug fluorescence merged with bright field images and lower row merged with nuclei

images after Hoechst staining. DMAG- 17-DMAG, Belin- belinostat, Dox- doxorubicin, Geld- geldanamycin, Pan- panobinostat. Images were taken 24h after addition of drugs.

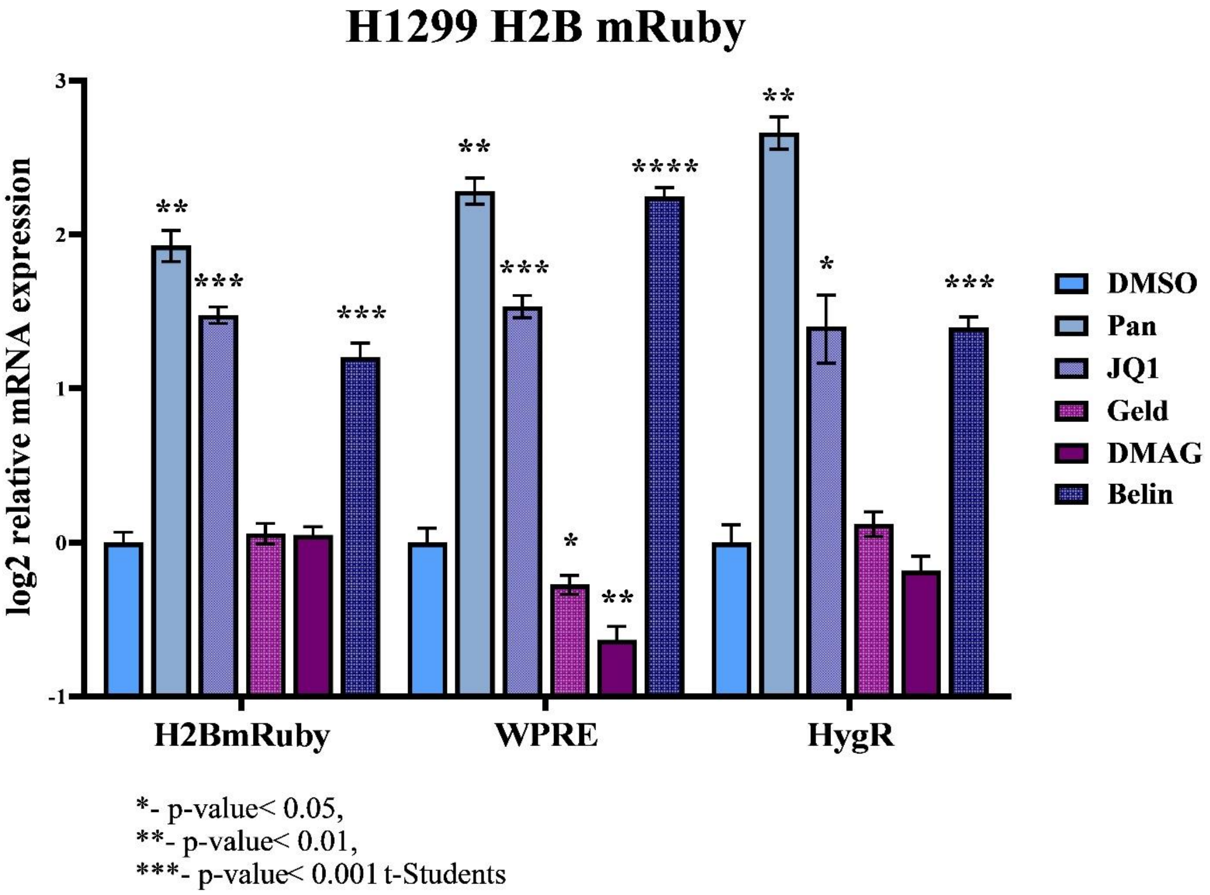

Figure S6. Lentiviral gene expression in H1299 cells. H1299 cells were cultivated at a density of 60,000 cells per well in a 6-well plate and treated to the IC50 concentrations of drugs, which lead to enhanced nuclear fluorescence. Expression of H2B-mRuby, WPRE, and hygromycin resistant gene (HygR) was measured 24 hours after treatment with DMSO, panobinostat (Pan), JQ-1, geldanamycin (Geld), 17-DMAG, belinostat (Belin) Real-time PCR was performed in triplicate and expression data was normalized to the expression levels of human GAPDH. Statistical significance was determined using Student’s t-test. *- $p<0.05$, **- $p<0.01$, ***- $p<0.001$

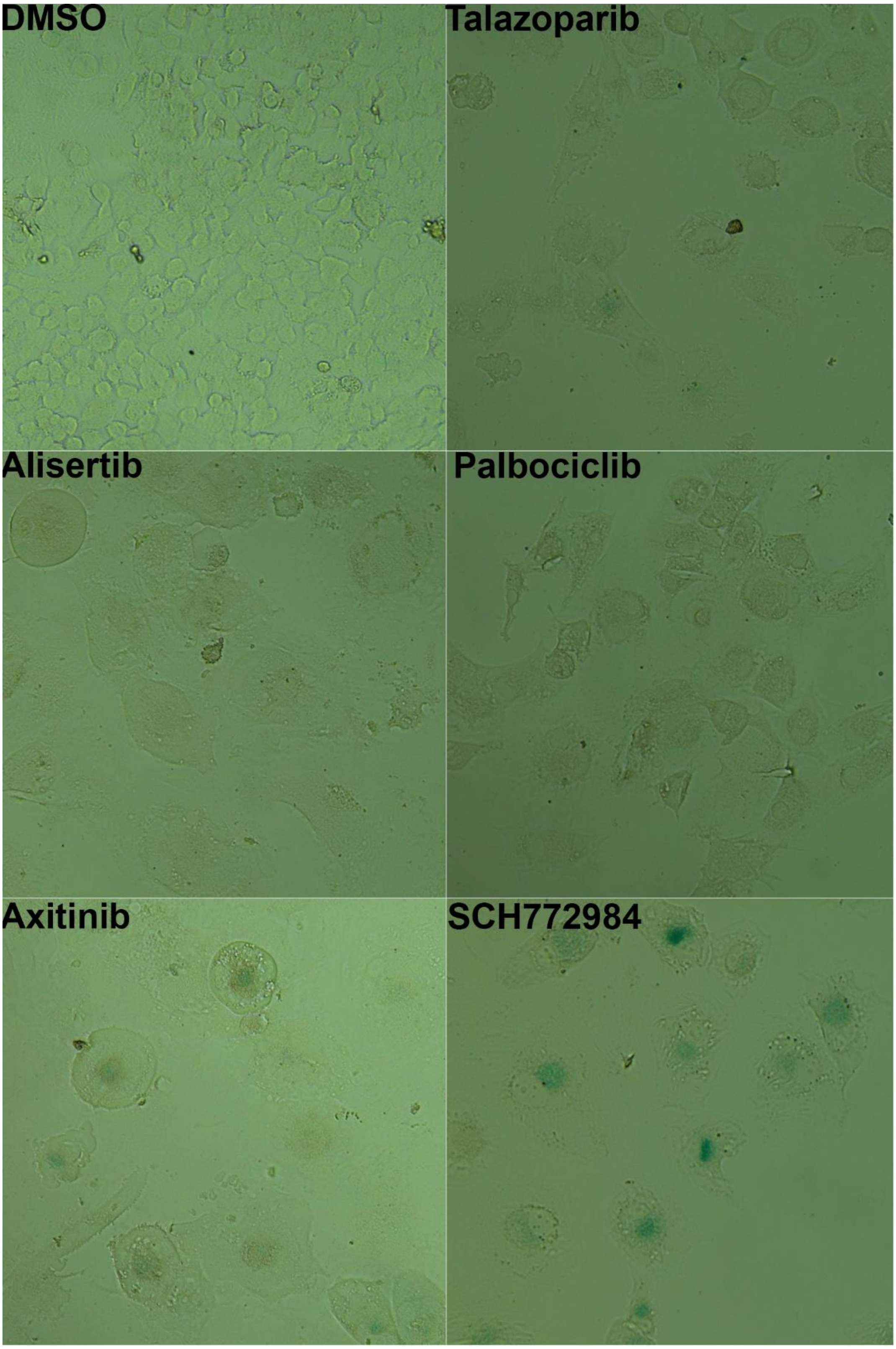

Figure S7. Senescence analysis in H1299 cells. Cells were stained for b-Gal activity (results into blue staining) 144h after addition of 25 uM talazoparib, 1000 nM alisertib, 20 uM axitinib, 10 uM palbociclib and 1 uM SCH772984. Treatment with SCH772984 was used as a positive control to induce senescence in cancer cells.

Table S1. Cell lines, reagents and drugs. Table contains data for reagents, cell lines, primers and drugs used in the study. Cell lines tab contains cultivation conditions for each cell line.

Table S2. IC50 and AUC values. Table contains calculated IC50 and AUC values for each combination of cell line, drug treatment and method (assay) used for cell viability measurement.

Table S3. Correlation between GDSC, CTRP and PRISM datasets. Table contains Spearman's correlation coefficients and p-values for each pairwise comparison.

Table S4. Differential changes in H2B-mRuby fluorescence. Table contains data for difference in mean H2B-mRuby2 normalized intensity compared to control treatment in DMSO, p-values obtained using Mann Whitney test and p-values adjusted for multiple comparisons using Benjamini-Hochberg correction (padj).
